# Congenital vertical talus: Treatment by reverse ponseti technique

**DOI:** 10.4103/0019-5413.41860

**Published:** 2008

**Authors:** Atul Bhaskar

**Affiliations:** BSES MG Global Hospital, Bombay Hospital Institute of Medical Sciences, Mumbai, India

**Keywords:** Casting, congenital vertical talus, conservative treatment

## Abstract

**Background:**

The surgery for idiopathic congenital vertical talus (CVT) can lead to stiffness, wound complications and under or over correction. There are sporadic literature on costing with mixed results. We describe our early experience of reverse ponseti technique.

**Materials and methods:**

Four cases (four feet) of idiopathic congenital vertical talus (CVT) which presented one month after birth were treated by serial manipulation and casting, tendoachilles tenotomy and percutaneous pinning of talonavicular joint. An average of 5.2 (range - four to six) plaster cast applications were required to correct the forefoot deformity. Once the talus and navicular were aligned based on the radiographic talus-first metatarsal axis, percutaneous fixation of the talo-navicular joint with a Kirschner wire, and percutaneous tendoachilles tenotomy under anesthesia was performed following which a cast was applied with the foot in slight dorsiflexion.

**Results:**

The mean follow-up period for the four cases was 8.5 months (6-12 months). At the end of the treatment all feet were supple and plantigrade but still using ankle foot orthosis (AFO). The mean talocalcaneal angle was 70 degrees before treatment and this reduced to 31 degrees after casting. The mean talar axis first metatasal base angle (TAMBA) angle was 60° before casting and this improved to 10.5°.

**Conclusion:**

Although our follow-up period is small, we would recommend early casting for idiopathic CVT along the same lines as the Ponseti technique for clubfoot except that the forces applied are in reverse direction. This early casting method can prevent extensive surgery in the future, however, a close vigil is required to detect any early relapse.

## INTRODUCTION

Congenital vertical talus (CVT) is a rigid flat foot deformity characterized by fixed hind foot equinus and an irreducible talonavicular dislocation. This deformity can either be idiopathic and isolated or can occur with other conditions such as: neural tube defects (myelomeningocoele and spina bifida occulta); neuromuscular disorders like cerebral palsy and anterior horn cell disease; malformation syndromes (Freeman-Sheldon and Marfan's) and in chromosomal aberrations (Down's syndrome).^1^

The foot has a typical rocker bottom appearance which is due to the hind foot equinus and markedly dorsiflexed and abducted forefoot. The tendoachilles is taut and ankle dorsiflexors are shortened due to the everted forefoot. Classical CVT is a rigid deformity and extensive surgery is usually required to restore foot alignment. Surgery can lead to stiffness, wound complications and under-correction and over-correction.^2^,^3^,^4^

The literature supporting early casting for CVT is sporadic and with mixed results, unlike in clubfoot where several reports testify the success of early cast treatment.^5^,^6^,^7^ There has been a recent interest in correcting CVT by serial casting to stretch the taut soft tissues and then performing minimal surgery which includes a percutaneous tendoachilles tenotomy and/or fractional lengthening of ankle dorsiflexors to achieve normal foot alignment. This treatment comprises serial casting in extreme equino-varus attitude, almost akin to creating a clubfoot deformity by reversing the Ponseti maneuver.^8^ Unlike in Ponseti technique where the emphasis is on abducting the forefoot to evert the os calcis, here one inverts and adducts the foot while applying upward pressure on the talar head.

We describe our early experience with using this technique in four consecutive cases with good success.

## MATERIALS AND METHODS

We analyzed four consecutive cases of CVT treated between 2006 and 2007. All cases were idiopathic in nature and involved three boys and one girl (four feet). Three children presented immediately after birth and one child presented after 30 days. All children were born at term of uneventful pregnancies. No other systemic or other congenital deformity was present in any child. Clinical examination revealed the classic deformity: an abducted forefoot with a convex sole and prominence of the talar head on the plantar aspect. The hind foot was fixed in equinus by the tight tendoachilles tendon. One girl had a clubfoot deformity on the opposite foot.

Radiographs in antero-posterior and stress plantar-flexion lateral view were taken to measure the talocalcaneal angle and the talar axis-first metatarsal base angle (TAMBA) in all children. Radiographs in the antero-posterior view and stress plantar-flexion view of the involved feet revealed fixed plantar-flexed attitude to talus and the calcaneus in persistent equinus. The TAMBA was used to exclude any oblique talus deformity which usually doesn't need any treatment.^8^

### Technique of casting and surgery

Serial casts were applied in the clinic after the child was adequately relaxed. No anesthesia or sedation was required for the procedure. The cast application was performed in two stages. Initially a below-knee cast is applied and the abducted forefoot is gently plantar-flexed and inverted with one hand while the other hand is used to manipulate the talus. A dorsally directed force is applied to the talar head to correct the plantar-flexed position. No attempt is made to correct the hind foot equinus. The inversion and adduction forces on the forefoot also correct, to some extent, the hind foot valgus. In the second stage the cast is extended above the knee. The knee is in about 90 degrees of flexion to prevent the cast from slippage and also maintain the achieved correction.

This procedure is repeated at a 7-10 days interval and usually four or five casts are required until the plantar-flexed talus is in line with the first ray as ascertained on a lateral radiograph.

**Figure 1:**
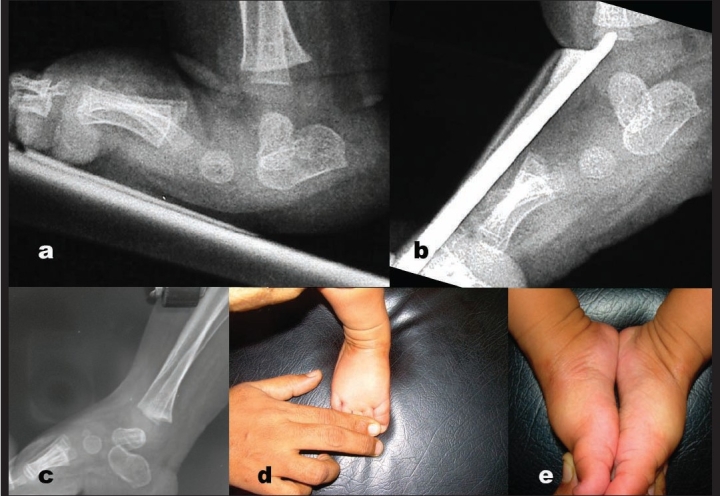
a) Lateral X-ray right foot in a child with congenital vertical talus shows irreducible talonavicular joint. b) Stress plantar flexion view of the same foot shows fixed plantar flexed talus. c) Follow up X-ray of the same foot shows reduction of talonavicular joint with the track of k-wire in talar body. d&e) Clinical photograph showing good correction of foot deformity and comparison with opposite foot.

Once this talar position is achieved then a closed pinning of the talonavicular joint is first performed under ‘C’ arm guidance under epidural anesthesia and sedation. A wire is introduced from the dorsum of the forefoot in line with the first ray (metatarsal) so as to pierce the navicular and this wire is advanced under radiographic control to hold the talonavicular joint. The wire placement is confirmed in both the antero-posterior (AP) and lateral planes. The wire should lie in the middle-third of the body of the talus to maintain the midfoot and forefoot alignment. Mini-open or a percutaneous tendoachilles tenotomy is performed to correct the hind foot equnius and valgus. If closed reduction is unsuccessful then the talo-navicular joint can be reduced under vision. Occasionally, the taut dorsal tendons (extensor digitorum longus, peroneus tertius) may need fractional lengthening. The foot is then put in cast in 5 degrees dorsiflexion for four weeks. The Kirschner wire is removed at four weeks, and measurements for a solid ankle foot orthosis (AFO) are made and the foot is recasted for another four weeks in 15 degrees of dorsiflexion.

The patient is fitted with a solid AFO at the end of eight weeks which is to be worn for 22 h per day. The parents are instructed to do regular stretching of soft tissues about 10 times a day. These include inverting the forefoot and plantar-flexion of the ankle to prevent any recurrence of tightness of ankle dorsiflexors.

## RESULTS

All children underwent serial casting after a mean interval of 14.2 days (7-30 days) from birth. The mean anteroposterior (AP) talocalcaneal angle was 70 degrees (60-80 degrees) and the mean TAMBA was 60 degrees (40-70 degrees) before manipulation.

A mean of 5.2 casts (four to six casts) were applied to achieve closed reduction of the talonavicular joint before the percutaneous fixation was attempted. No foot required open release and fractional lengthening of tendons in this small series. The total time required for treatment was approximately 13-14 weeks, depending on the number of cast changes required to restore the plantar-flexed talus in line with the first ray. The mean follow-up period was 8.5 months (range 6-12 months) and at the final follow-up all feet were supple and plantigrade. The mean final AP talocalcaneal angle was 31° (24-36°) degrees and the mean lateral TAMBA was 10°5. (6-15°).

The ankle range of motion was measured clinically and ankle dorsiflexion averaged 27° (25°-30°) and plantar flexion averaged 17° (15°-20°). Although it was possible to determine the subtalar motion this was not recorded as it is technically difficult to gauge motion in such small feet. One child walks unsupported and three stand with support. In all children, the foot correction was achieved before walking age and all are using a custom-made AFO.

No complications were noted during treatment in this short follow-up period. All children are still under review. No scoring system was used to assess the results in view of the short follow-up period.

Limitations of this study: It is a single-author review and observed study; the follow-up period is short with only 4 cases to make any major treatment suggestions.

## DISCUSSION

The exact etiology of CVT remains an enigma and it may be due to an arrest in the prenatal development of the foot. Paralysis and contracture of the soft tissue also have been implicated in its pathogenesis.^9^ Congenital vertical talus can occur as an isolated deformity, but in more than 50% cases a secondary cause is usually implicated.

The pathological anatomy involves dislocation of the talonavicular articulation with the os calcis also rotated in plantar-flexion. The navicular bone is displaced onto the dorsolateral aspect of the talar head. The ligaments and capsule on the plantar aspect are stretched whereas those on the dorsolateral surfaces are contracted. The long toe extensors and peronei are also foreshortened and bowstring occurs across the midfoot.

Standard AP and lateral radiographs, and forced plantar flexion and dorsiflexion radiographs are required to confirm the diagnosis and to assess reducibility of the deformity. Since the navicular is unossified at birth, the relationship between the talus and navicular is delineated by drawing lines through the longitudinal axis of talus and first metatarsal. In CVT the longitudinal axis of talus is almost parallel to tibia because of the extreme plantar-flexion of talus. Also, this position does not change when the radiograph is taken in maximum plantar-flexion. The longitudinal axis of the first metatarsal passes dorsal to the head of talus. The angular relationship between talus and first metatarsal axis has been called the TAMBA (Talus axis and metatarsal base axis) angle. In oblique view the talus is plantar flexed like in the vertical talus with foot in neutral position but on plantar-flexion radiograph the long axis of talus and long axis of first metatarsal line up, thus a fixed deformity is ruled out. Hence, it is important to take a stress plantar-flexion radiograph in every suspected case of vertical talus.^10^

In a true CVT, the deformity is irreducible. After a few initial casts to stretch the soft tissue, surgery is usually performed at around six to eight months of age. Two-incision approach or a Cincinnati incision is used to perform a comprehensive posterolateral and medial release. Coleman described a two-stage operation to restore the talonavicular alignment. The first stage involved correcting the dorsolateral soft tissue contracture and in the second stage the hind foot equnius was corrected and the tibialis posterior tendon was advanced to the plantar aspect of the navicular.^2^ Duncan and Fixsen have used the tibialis anterior tendon assay sling around the talus neck to maintain the correction.^11^ Both these procedures are used in older children when the deformities are more rigid and unyielding to plaster cast application. A pin across the talonavicular joint is passed after an open release and usually kept for six weeks. After surgery, casting and prolonged splinting is still required to prevent any recurrence.

Serial cast treatment to correct CVT has met with limited success. Eraltug reported 11 cases that were corrected by cast application. Seven cases had recurrence of which four had a neuromuscular pathology.^12^ Storen also described cast correction for CVT.^13^ He did not provide the technical details about casting but reported two failures out of five cases.

Recently Dobbs *et al.*, reported successful results with their technique of cast treatment and limited surgical intervention.^8^ Of the 11 cases (19 feet), three had a recurrence (six feet). In the remaining cases, where the talonavicular articulation was pinned percutaneously, no recurrence was observed at the end of two years. We have used the same principles as described by Dobbs *et al*.

There are a few limitations of our study. There are only four cases and it is a single-author experience with a short follow-up. This technique may be an alternative treatment to achieve correction of CVT without major soft tissue surgery in CVT of the idiopathic type.
